# Obesity Prevalence Among Adults Living in Metropolitan and Nonmetropolitan Counties — United States, 2016

**DOI:** 10.15585/mmwr.mm6723a1

**Published:** 2018-06-15

**Authors:** Elizabeth A. Lundeen, Sohyun Park, Liping Pan, Terry O’Toole, Kevin Matthews, Heidi M. Blanck

**Affiliations:** ^1^Division of Nutrition, Physical Activity, and Obesity, National Center for Chronic Disease Prevention and Health Promotion, CDC; ^2^Division of Population Health, National Center for Chronic Disease Prevention and Health Promotion, CDC.

Approximately 46 million persons (14%) in the United States live in nonmetropolitan counties.* Compared with metropolitan residents, nonmetropolitan residents have a higher prevalence of obesity-associated chronic diseases such as diabetes (*1*), coronary heart disease (*1*), and arthritis (*2*). The 2005–2008 National Health and Nutrition Examination Survey (NHANES) found a significantly higher obesity prevalence among adults in nonmetropolitan (39.6%) than in metropolitan (33.4%) counties (*3*). However, this difference has not been examined by state. Therefore, CDC examined state-level 2016 Behavioral Risk Factor Surveillance System (BRFSS) data and found that the prevalence of obesity (body mass index [BMI] ≥30 kg/m^2^) was 34.2% among U.S. adults living in nonmetropolitan counties and 28.7% among those living in metropolitan counties (p<0.001). Obesity prevalence was significantly higher among nonmetropolitan county residents than among metropolitan county residents in all U.S. Census regions, with the largest absolute difference in the South (5.6 percentage points) and Northeast (5.4 percentage points). In 24 of 47 states, obesity prevalence was significantly higher among persons in nonmetropolitan counties than among those in metropolitan counties; only in Wyoming was obesity prevalence higher among metropolitan county residents than among nonmetropolitan county residents. Both metropolitan and nonmetropolitan counties can address obesity through a variety of policy and environmental strategies to increase access to healthier foods and opportunities for physical activity (*4*).

BRFSS is a state-based, random-digit–dialed telephone survey of U.S. adults aged ≥18 years, conducted annually by CDC and state and territorial health departments to monitor health conditions and related behaviors.^†^ BRFSS uses multistage, stratified sampling to select a representative sample of the noninstitutionalized adult population in 50 states, the District of Columbia (DC), and selected U.S. territories. In 2016, using combined landline and cell phone data across all states, the median response rate was 47.0%, which was calculated using rates from the American Association of Public Opinion Research.^§^ Self-reported weight and height were used to calculate BMI (weight [kg]/height [m]^2^); obesity was defined as BMI ≥30 kg/m^2^.^¶^ Among 477,665 respondents, 39,186 (8.2%) were excluded, including 36,848 with missing BMI values and 2,338 with implausible BMI values, leaving a final analytic sample of 438,479 adults from 50 states and DC. Unadjusted obesity prevalence is presented overall and by sociodemographic characteristics (age, sex, race/ethnicity, education, income, and employment status), state, and four U.S. Census regions and nine divisions: Northeast region (New England and Middle Atlantic divisions), Midwest region (East North Central and West North Central divisions), South region (South Atlantic, East South Central, and West South Central divisions), and West region (Mountain and Pacific divisions).**

Using 2010 Census data, CDC’s National Center for Health Statistics (NCHS) developed an Urban-Rural Classification Scheme for Counties,^††^ which specified six county types; for this analysis, to ensure sufficient sample size for regional and state-level comparisons, counties were collapsed into two categories: metropolitan (large central metro, large fringe metro, medium metro, and small metro) and nonmetropolitan (micropolitan and noncore). In this analysis, the nonmetropolitan designation was used to classify counties with small populations (<50,000). Rhode Island, New Jersey, Delaware, and DC do not have nonmetropolitan counties; for these jurisdictions, obesity prevalence was calculated for adults living in metropolitan counties only. U.S. territories were excluded because the NCHS classification scheme does not include them. Unadjusted obesity prevalence was stratified by metropolitan and nonmetropolitan status. Differences in obesity prevalence between adults living in metropolitan and nonmetropolitan counties were examined using multivariable logistic regression, controlling for age, sex, and race/ethnicity within levels of the sociodemographic characteristics, states, and Census regions and divisions (statistically significant at p<0.05). All analyses accounted for complex survey design and sampling weights.

In 2016, overall obesity prevalence was 29.6% and was highest among persons residing in the South (32.0%) and Midwest (31.4%) regions and the East South Central (35.3%) and West South Central (33.9%) divisions (Table 1). Overall, obesity prevalence was significantly higher among adults living in nonmetropolitan counties (34.2%) than among those living metropolitan counties (28.7%) (p<0.001), and the same was found in all Census regions and Census divisions. Among Census regions, the largest difference in obesity prevalence between persons living in nonmetropolitan and metropolitan counties was in the South (5.6 percentage points) and Northeast (5.4 percentage points); among Census divisions, the largest difference in obesity prevalence between nonmetropolitan and metropolitan residents was in the Middle Atlantic division (6.6 percentage points). Obesity prevalence was also significantly higher among nonmetropolitan county residents than among metropolitan county residents for all sociodemographic categories except Hispanics and persons with less than a high school education.

**TABLE 1:** Prevalence of self-reported obesity among adults (aged ≥18 years) by respondent characteristics and metropolitan/nonmetropolitan status — Behavioral Risk Factor Surveillance System, 50 states and the District of Columbia, 2016

Characteristic	No. of respondents	Unadjusted adult obesity prevalence–weighted % (95% CI)*		
		Total	Metropolitan^†^	Nonmetropolitan^†^
**Total**	**438,479**	**29.6 (29.3–29.8)**	**28.7 (28.4–29.0)^§^**	**34.2 (33.6–34.8)^§^**
**Age group (yrs)^¶^**				
18–24	23,734	17.3 (16.5–18.1)	16.5 (15.6–17.4)^§^	22.2 (20.3–24.2)^§^
25–34	42,706	27.2 (26.5–27.9)	26.4 (25.6–27.2)^§^	32.5 (30.8–34.3)^§^
35–44	48,951	33.1 (32.3–33.8)	32.0 (31.2–32.9)^§^	39.6 (38.0–41.2)^§^
45–54	68,854	35.1 (34.4–35.8)	34.0 (33.2–34.8)^§^	40.8 (39.4–42.3)^§^
55–64	96,566	34.2 (33.6–34.8)	33.4 (32.7–34.1)^§^	38.0 (36.9–39.2)^§^
≥65	157,668	28.0 (27.5–28.5)	27.5 (26.9–28.1)^§^	30.1 (29.3–31.0)^§^
Sex**				
Male	198,440	29.6 (29.2–30.0)	28.8 (28.3–29.2)^§^	34.4 (33.6–35.2)^§^
Female	240,000	29.5 (29.1–29.9)	28.7 (28.2–29.1)^§^	34.0 (33.2–34.8)^§^
Race/Ethnicity^¶,^**				
White, non-Hispanic	341,192	28.6 (28.3–28.9)	27.5 (27.2–27.9)^§^	33.2 (32.6–33.8)^§^
Black, non-Hispanic	35,091	38.3 (37.4–39.3)	37.7 (36.7–38.7)^§^	44.2 (41.7–46.7)^§^
Hispanic, any race	28,666	33.1 (32.1–34.1)	32.9 (31.9–33.9)	36.0 (32.6–39.5)
Other, non-Hispanic	26,954	18.2 (17.3–19.2)	16.8 (15.8–17.8)^§^	33.2 (31.2–35.3)^§^
Education^¶,^**				
<High school	32,325	35.5 (34.5–36.5)	35.4 (34.3–36.6)	35.9 (34.0–37.8)
High school	123,241	32.3 (31.8–32.8)	31.5 (30.9–32.1)^§^	35.6 (34.7–36.5)^§^
Some college	120,735	31.0 (30.5–31.5)	30.3 (29.7–30.9)^§^	34.7 (33.7–35.7)^§^
College graduate	161,309	22.2 (21.9–22.6)	21.5 (21.1–21.9)^§^	28.8 (27.9–29.7)^§^
Annual household income^¶,^**				
<$25,000	99,244	34.1 (33.5–34.7)	33.4 (32.7–34.2)^§^	37.1 (35.9–38.2)^§^
$25,000–49,999	95,553	31.9 (31.3–32.6)	31.1 (30.3–31.8)^§^	35.9 (34.7–37.1)^§^
$50,000–74,999	61,211	31.1 (30.3–31.8)	30.2 (29.4–31.1)^§^	35.4 (34.0–36.8)^§^
≥$75,000	120,901	25.4 (24.9–25.9)	24.8 (24.3–25.3)^§^	30.9 (29.8–32.1)^§^
Employment status^¶,^**				
Employed	215,226	29.0 (28.6–29.4)	28.2 (27.8–28.6)^§^	34.1 (33.3–34.9)^§^
Out of work	17,009	32.9 (31.6–34.3)	32.4 (30.9–34.0)^§^	35.8 (33.1–38.7)^§^
Homemaker	22,372	29.0 (27.7–30.3)	28.4 (27.0–29.9)^§^	32.0 (29.5–34.7)^§^
Student	11,277	15.2 (14.1–16.3)	14.8 (13.6–16.0)^§^	18.8 (16.2–21.7)^§^
Retired	136,638	29.1 (28.5–29.6)	28.6 (28.0–29.2)^§^	31.2 (30.3–32.2)^§^
Unable to work	33,534	45.8 (44.8–46.9)	45.5 (44.2–46.8)^§^	47.1 (45.2–49.1)^§^
**Census region^¶,††^**				
Northeast	88,335	26.9 (26.3–27.5)	26.4 (25.8–27.0)^§^	31.8 (30.4–33.2)^§^
Midwest	106,697	31.4 (30.9–31.9)	30.5 (29.9–31.2)^§^	34.2 (33.3–35.1)^§^
South	146,919	32.0 (31.5–32.5)	31.0 (30.4–31.6)^§^	36.6 (35.6–37.6)^§^
West	96,528	26.0 (25.4–26.6)	25.7 (25.1–26.4)^§^	28.6 (27.5–29.7)^§^
**Census division^¶,††^**				
New England	43,889	25.4 (24.7–26.1)	25.0 (24.2–25.8)^§^	28.7 (27.4–30.0)^§^
Middle Atlantic	44,446	27.4 (26.7–28.2)	26.9 (26.1–27.7)^§^	33.5 (31.5–35.6)^§^
East North Central	42,215	31.8 (31.1–32.5)	31.0 (30.2–31.8)^§^	34.9 (33.5–36.3)^§^
West North Central	64,482	30.6 (30.0–31.2)	29.3 (28.5–30.1)^§^	33.3 (32.4–34.2)^§^
South Atlantic	93,367	29.9 (29.3–30.4)	29.1 (28.5–29.7)^§^	35.3 (33.9–36.7)^§^
East South Central	26,587	35.3 (34.4–36.2)	34.5 (33.3–35.6)^§^	36.9 (35.6–38.1)^§^
West South Central	26,965	33.9 (32.7–35.2)	33.1 (31.7–34.5)^§^	37.8 (35.4–40.3)^§^
Mountain	57,788	26.2 (25.6–26.8)	26.0 (25.3–26.7)^§^	27.2 (26.3–28.1)^§^
Pacific	38,740	25.9 (25.0–26.7)	25.6 (24.7–26.4)^§^	30.3 (28.1–32.6)^§^

**Abbreviation:** CI = confidence interval.

* Obesity defined as having a body mass index ≥30 kg/m^2^ determined by self-reported weight and height.

^†^ Based on National Center for Health Statistics Urban-Rural Classification Scheme for Counties. Metropolitan includes large central metro, large fringe metro, medium metro, and small metro categories. Nonmetropolitan includes micropolitan and noncore categories.

^§^ Significant difference in the prevalence of obesity between metropolitan and nonmetropolitan areas at the p<0.05 level. Determined using multivariable logistic regression within levels of the sociodemographic and geographic characteristics to control for age, sex, and race/ethnicity.

^¶^ Significant difference in the prevalence of obesity across levels of the characteristic at the p<0.05 level using Chi-square test.

** Missing data: sex (n = 39; 0.009%), race/ethnicity (n = 6,576; 1.5%), education (n = 869; 0.2%), income (n = 61,570; 14.0%), and employment status (n = 2,423; 0.6%).

^††^ The United States Census Bureau defines four census regions and nine census divisions: Northeast region (New England and Middle Atlantic divisions), Midwest region (East North Central and West North Central divisions), Southern region (South Atlantic, East South Central, and West South Central divisions), and Western region (Mountain and Pacific divisions).

Among adults living in nonmetropolitan counties, obesity prevalence ranged from 20.8% in Colorado to 39.1% in Louisiana; among those living in metropolitan counties, prevalence ranged from 22.5% in Colorado to 36.9% in West Virginia. (Table 2). In 24 (51%) of the 47 states with both metropolitan and nonmetropolitan counties, obesity prevalence was significantly higher among adults living in nonmetropolitan counties than among those living in metropolitan counties; in 22 (47%) states, no difference was observed. Wyoming was the only state where obesity prevalence was significantly higher among metropolitan county residents (32.8%) than among nonmetropolitan residents (25.4%; p = 0.002).

**TABLE 2:** Prevalence of self-reported obesity among adults (aged ≥18 years) by state and metropolitan/nonmetropolitan status — Behavioral Risk Factor Surveillance System, 50 states and the District of Columbia, 2016

Census division^†^/State	No. of respondents	Unadjusted adult obesity prevalence–weighted % (95% CI)*	
		Metropolitan^§^	Nonmetropolitan^§^
**New England**			
Connecticut	9,960	25.9 (24.7–27.1)	28.1 (22.7–34.2)
Maine	9,554	29.3 (27.3–31.3)	30.9 (29.1–32.7)
Massachusetts	7,480	23.6 (22.2–24.9)	24.4 (16.9–34.0)
New Hampshire	5,888	26.0 (23.8–28.2)	27.6 (25.4–29.9)
Rhode Island	4,936	26.6 (24.9–28.4)	^—¶^
Vermont	6,071	24.1 (21.3–27.1)**	28.7 (26.9–30.6)**
**Middle Atlantic**			
New Jersey	6,810	27.4 (25.7–29.1)	^—¶^
New York	31,269	24.9 (23.9–26.0)**	33.0 (31.6–34.5)**
Pennsylvania	6,367	29.7 (28.1–31.4)**	33.9 (30.4–37.5)**
**East North Central**			
Illinois	4,518	31.0 (29.2–32.9)**	35.7 (31.0–40.6)**
Indiana	10,319	32.0 (30.6–33.5)	33.9 (31.3–36.7)
Michigan	11,130	31.6 (30.4–32.9)**	36.0 (33.7–38.5)**
Ohio	11,455	30.7 (29.2–32.3)**	34.4 (32.1–36.8)**
Wisconsin	4,793	29.1 (27.0–31.3)**	34.4 (31.6–37.3)**
**West North Central**			
Iowa	6,645	31.4 (29.4–33.5)	32.7 (30.7–34.8)
Kansas	10,947	29.9 (28.5–31.3)**	33.7 (32.0–35.5)**
Minnesota	15,420	26.5 (25.6–27.5)**	31.7 (30.1–33.2)**
Missouri	6,578	30.5 (28.4–32.6)**	34.9 (32.1–37.9)**
Nebraska	14,173	30.8 (29.1–32.6)**	34.2 (32.9–35.5)**
North Dakota	5,348	30.5 (28.2–32.9)	33.4 (31.2–35.6)
South Dakota	5,371	27.0 (23.9–30.5)**	31.8 (29.2–34.5)**
**South Atlantic**			
Delaware	3,702	30.7 (28.7–32.8)	^—¶^
District of Columbia	3,479	22.6 (20.9–24.3)	^—¶^
Florida	33,186	27.2 (26.1–28.2)**	34.9 (32.6–37.2)**
Georgia	4,884	30.8 (28.9–32.8)	34.0 (30.3–37.9)
Maryland	16,701	29.8 (28.7–30.9)**	35.1 (32.0–38.3)**
North Carolina	5,984	31.1 (29.5–32.9)	34.1 (31.4–37.0)
South Carolina	10,503	31.2 (29.8–32.7)**	37.8 (35.1–40.6)**
Virginia	8,293	27.7 (26.3–29.1)**	36.1 (33.2–39.1)**
West Virginia	6,635	36.9 (35.2–38.7)	38.8 (36.6–41.0)
**East South Central**			
Alabama	6,526	35.6 (33.8–37.5)	36.0 (33.1–38.9)
Kentucky	9,583	32.1 (30.2–34.0)**	36.9 (34.7–39.2)**
Mississippi	4,821	36.5 (33.4–39.7)	37.9 (35.7–40.1)
Tennessee	5,657	34.3 (32.1–36.6)	36.4 (33.6–39.3)
**West South Central**			
Arkansas	4,859	35.4 (32.2–38.8)	36.1 (32.6–39.7)
Louisiana	4,868	34.8 (32.5–37.3)	39.1 (34.7–43.7)
Oklahoma	6,449	30.8 (28.8–32.8)**	36.3 (33.9–38.8)**
Texas	10,789	32.9 (31.0–34.8)**	38.7 (34.3–43.2)**
**Mountain**			
Arizona	10,033	28.8 (27.2–30.4)	33.6 (29.1–38.4)
Colorado	13,637	22.5 (21.5–23.5)	20.8 (19.0–22.8)
Idaho	4,880	26.3 (23.9–28.8)	29.6 (27.0–32.4)
Montana	5,483	25.9 (23.1–29.0)	25.3 (23.3–27.3)
Nevada	3,981	25.1 (23.1–27.3)**	32.1 (28.6–35.9)**
New Mexico	5,531	27.0 (24.7–29.4)**	31.1 (28.7–33.6)**
Utah	10,043	25.4 (24.2–26.7)	24.9 (22.7–27.2)
Wyoming	4,200	32.8 (29.0–36.9)**	25.4 (23.1–27.8)**
**Pacific**			
Alaska	2,739	30.9 (27.1–35.0)	32.4 (28.8–36.4)
California	10,352	25.0 (24.0–26.1)	24.2 (19.2–30.0)
Hawaii	7,659	23.3 (21.8–24.9)**	26.1 (23.5–28.8)**
Oregon	5,000	27.4 (25.8–29.1)**	35.1 (31.5–38.8)**
Washington	12,990	27.8 (26.8–28.9)**	35.3 (32.3–38.4)**

**Abbreviation:** CI = confidence interval.

* Obesity defined as having a body mass index ≥30 kg/m^2^, determined by self-reported weight and height.

^†^ The United States Census Bureau defines nine census divisions within four regions: Northeast region (New England and Middle Atlantic divisions), Midwest region (East North Central and West North Central divisions), Southern region (South Atlantic, East South Central, and West South Central divisions), and Western region (Mountain and Pacific divisions).

^§^ Based on National Center for Health Statistics Urban-Rural Classification Scheme for Counties. Metropolitan includes large central metro, large fringe metro, medium metro, and small metro categories. Nonmetropolitan includes micropolitan and noncore categories.

^¶^ Data not available because state does not have counties in the nonmetropolitan classification.

** Significant difference in the prevalence of obesity between metropolitan and nonmetropolitan areas at the p<0.05 level. Within states, differences in obesity prevalence between metropolitan and nonmetropolitan areas were determined using multivariable logistic regression, controlling for age, sex, and race/ethnicity.

## Discussion

In this study, obesity prevalence was significantly higher among adults living in nonmetropolitan counties than among those living in metropolitan counties, overall, in all Census regions, all Census divisions, and in approximately half of states with both county types. Across regions and divisions, this disparity in obesity prevalence was highest in the South and Northeast regions and the Middle Atlantic division. With the exception of Hispanics and persons with less than a high school education, the higher obesity prevalence among nonmetropolitan residents was observed in all sociodemographic groups.

These findings are consistent with those previously reported using 2005–2008 NHANES data, which documented higher overall obesity prevalence among adults living in nonmetropolitan versus metropolitan counties of the United States (*3*), and expand the understanding of this health disparity by highlighting differences across states and regions. Research has documented differences between adults living in nonmetropolitan and metropolitan counties in health behaviors and community factors, which could influence obesity risk (*5*–*7*). An analysis of 2013 BRFSS data found that adults living in U.S. nonmetropolitan counties were less physically active and less likely to meet physical activity recommendations than their metropolitan counterparts (*5*). Data from 2011 indicated that across all regions, adults living in rural areas were less likely to have access to healthier food retailers (supermarkets, large grocery stores, and fruit/vegetable specialty stores) than were those living in urban areas (*6*). In addition, several social determinants of health that are risk factors for obesity, such as persistent poverty and food insecurity (*7*), are more prevalent in rural than in urban areas.^§§^^,^^¶¶^

In this analysis, the highest obesity prevalence and the greatest disparity in prevalence between persons living in nonmetropolitan and metropolitan counties were in the South Census region. One possible contributing factor is the high rate of persistent poverty in the South, which also is affected by the largest difference in poverty rate between metropolitan and nonmetropolitan county residents.^¶¶^

The findings in this report are subject to at least two limitations. First, data are self-reported, and self-reported weight and height data underestimate BMI values, particularly among persons with a higher BMI (*8*). It is not known whether self-reporting bias is comparable across regions and between metropolitan and nonmetropolitan counties. Second, to ensure sufficient sample size for regional and state-level comparisons, the nonmetropolitan classification was used to designate counties with small populations (<50,000 persons). The literature on rural obesity disparities and prevention strategies uses various methods to define rural areas, some of which might differ in population size from the nonmetropolitan designation used in this paper.

CDC recommends 24 obesity-prevention policy and environmental strategies (*4*). Two systematic reviews summarized the relevance and effectiveness of these strategies in rural areas and identified how these strategies could be adapted for rural settings (*9*,*10*). One nutrition-related obesity prevention strategy, increasing the availability of healthier food and beverage choices, is challenging to implement in rural areas because of the long distances between food suppliers and retailers and between retailers and consumers, which can influence food cost and the availability of fresh foods. Approaches to overcoming this challenge include strengthening networks between food producers, distributors, and retail food outlets, as well as reducing the distance customers need to travel, for example, by increasing access to nearby farmers’ markets (*9*). The 2018 CDC State Indicator Report on Fruits and Vegetables also highlights approaches to increase the purchase, supply, and demand of fruits and vegetables in states and communities across the United States.*** Other approaches include working with schools and worksites to develop nutrition-related policies and forming strong partnerships with groups such as the Cooperative Extension Service to promote federal food and nutrition assistance program benefits (*9*). 

Strategies to increase physical activity in rural areas should take into consideration geographic dispersion, transportation challenges, and limitations on community resources that might not be present in urban areas (*10*). Strategies that have been implemented in rural settings include improving community access to public buildings (e.g., school facilities) after hours for physical activity purposes; improving infrastructure and land use design to support walking and other physical activity (e.g., bicycle paths, paved sidewalks, and outdoor public recreation facilities); promoting existing places for physical activity with improved signage; enhancing physical education in schools; and implementing worksite policies to promote physical activity (*10*). The data in this report can serve as a resource for states seeking to reduce obesity disparities in nonmetropolitan counties through strategies to increase physical activity and healthier eating.

**Summary**

**What is already known about this topic?**

National estimates from a decade ago found a higher prevalence of obesity among adults living in nonmetropolitan counties than among those living in metropolitan counties.

**What is added by this report?**

Analysis of 2016 Behavioral Risk Factor Surveillance System data found a higher obesity prevalence among adults in nonmetropolitan counties than among those in metropolitan counties. The greatest differences in obesity prevalence between nonmetropolitan and metropolitan residents were in the South (5.6 percentage points) and Northeast (5.4 percentage points).

**What are the implications for public health practice?**

Both nonmetropolitan and metropolitan counties can address obesity through a variety of policy and environmental strategies to increase access to healthier foods and opportunities for physical activity.

## Footnotes

* https://www.ers.usda.gov/webdocs/publications/80894/eib-162.pdf?v=42684.

† https://www.cdc.gov/brfss/annual_data/annual_2016.html.

§ https://www.cdc.gov/brfss/annual_data/2016/pdf/2016-sdqr.pdf.

¶ https://www.nhlbi.nih.gov/files/docs/guidelines/ob_gdlns.pdf.

** https://www.census.gov/geo/reference/gtc/gtc_census_divreg.html.

†† https://www.cdc.gov/nchs/data_access/urban_rural.htm.

§§ https://www.ers.usda.gov/webdocs/publications/79761/err-215.pdf?v=42636.

¶¶ https://www.ers.usda.gov/topics/rural-economy-population/rural-poverty-well-being/.

*** https://www.cdc.gov/nutrition/data-statistics/2018-state-indicator-report-fruits-vegetables.html.

## References

[1] O’Connor A, Wellenius G. Rural-urban disparities in the prevalence of diabetes and coronary heart disease. Public Health 2012;126:813–20. 10.1016/j.puhe.2012.05.029 22922043

[2] Boring MA, Hootman JM, Liu Y, Prevalence of arthritis and arthritis-attributable activity limitation by urban-rural county classification—United States, 2015. MMWR Morb Mortal Wkly Rep 2017;66:527–32. 10.15585/mmwr.mm6620a2 28542117 PMC5657875

[3] Befort CA, Nazir N, Perri MG. Prevalence of obesity among adults from rural and urban areas of the United States: findings from NHANES (2005–2008). J Rural Health 2012;28:392–7. 10.1111/j.1748-0361.2012.00411.x 23083085 PMC3481194

[4] Khan LK, Sobush K, Keener D, . Recommended community strategies and measurements to prevent obesity in the United States. MMWR Recomm Rep 2009;58(No. RR-7). 19629029

[5] Matthews KA, Croft JB, Liu Y, Health-related behaviors by urban-rural county classification—United States, 2013. MMWR Surveill Summ 2017;66(No. SS-5). 10.15585/mmwr.ss6605a1 28151923 PMC5829834

[6] Grimm KA, Moore LV, Scanlon KS. Access to healthier food retailers—United States, 2011. MMWR Suppl 2013;62:20–6. 24264485

[7] Bhattacharya J, Currie J, Haider S. Poverty, food insecurity, and nutritional outcomes in children and adults. J Health Econ 2004;23:839–62. 10.1016/j.jhealeco.2003.12.008 15587700

[8] Stommel M, Schoenborn CA. Accuracy and usefulness of BMI measures based on self-reported weight and height: findings from the NHANES & NHIS 2001–2006. BMC Public Health 2009;9:421. 10.1186/1471-2458-9-421 19922675 PMC2784464

[9] Calancie L, Leeman J, Jilcott Pitts SB, Nutrition-related policy and environmental strategies to prevent obesity in rural communities: a systematic review of the literature, 2002–2013. Prev Chronic Dis 2015;12:140540. 10.5888/pcd12.140540 25927605 PMC4416478

[10] Umstattd Meyer MR, Perry CK, Sumrall JC, Physical activity–related policy and environmental strategies to prevent obesity in rural communities: a systematic review of the literature, 2002–2013. Prev Chronic Dis 2016;13:150406. 10.5888/pcd13.150406 26741997 PMC4707945

